# Sex-dependent gut microbiota-brain-cognition associations: a multimodal MRI study

**DOI:** 10.1186/s12883-023-03217-3

**Published:** 2023-04-27

**Authors:** Shujun Zhang, Huanhuan Cai, Chunli Wang, Jiajia Zhu, Yongqiang Yu

**Affiliations:** 1 Department of Radiology, Affiliated Hospital of Jining Medical University, Jining, 272007 China; 2 Department of Radiology, The First Affiliated Hospital of Anhui Medical University, No. 218, Jixi Road, Shushan District, Hefei, 230022 China; 3 Research Center of Clinical Medical Imaging, Anhui Province, Hefei, 230032 China; 4 Anhui Provincial Institute of Translational Medicine, Hefei, 230032 China; 5 Department of Clinical Laboratory, The First Affiliated Hospital of Anhui Medical University, Hefei, 230022 China

**Keywords:** Gut microbiota, Brain, Sex, Magnetic resonance imaging, Cognition

## Abstract

**Background:**

There is bidirectional communication between the gut microbiota and the brain. Empirical evidence has demonstrated sex differences in both the gut microbiome and the brain. However, the effects of sex on the gut microbiota-brain associations have yet to be determined. We aim to elucidate the sex-specific effects of gut microbiota on brain and cognition.

**Methods:**

One hundred fifty-seven healthy young adults underwent brain structural, perfusion, functional and diffusion MRIs to measure gray matter volume (GMV), cerebral blood flow (CBF), functional connectivity strength (FCS) and white matter integrity, respectively. Fecal samples were collected and 16S amplicon sequencing was utilized to assess gut microbial diversity. Correlation analyses were conducted to test for sex-dependent associations between microbial diversity and brain imaging parameters, and mediation analysis was performed to further characterize the gut microbiota-brain-cognition relationship.

**Results:**

We found that higher gut microbial diversity was associated with higher GMV in the right cerebellum VI, higher CBF in the bilateral calcarine sulcus yet lower CBF in the left superior frontal gyrus, higher FCS in the bilateral paracentral lobule, and lower diffusivity in widespread white matter regions in males. However, these associations were absent in females. Of more importance, these neuroimaging biomarkers significantly mediated the association between gut microbial diversity and behavioral inhibition in males.

**Conclusions:**

These findings highlight sex as a potential influential factor underlying the gut microbiota-brain-cognition relationship, and expose the gut microbiota as a biomarker-driven and sex-sensitive intervention target for mental disorders with abnormal behavioral inhibition.

**Supplementary Information:**

The online version contains supplementary material available at 10.1186/s12883-023-03217-3.

## Introduction

Increasing evidence endorses the notion that there is bidirectional communication between the gut microbiota and the central nervous system through the microbiota-gut-brain axis [1–3]. On one hand, the gut microbiome can influence the development, aging and neurodegeneration of the brain, which in turn could have consequences for subsequent behavior [4]. A plausible explanation lies in the fact that gut bacteria are capable of synthesizing and releasing various metabolites including neuropeptides and neurotransmitters [5], which might signal to the brain via nervous, endocrine, and immune systems. On the other hand, the brain can impact the gut microbiota through the autonomic nervous system, by modulating gut functions (e.g., motility, intestinal transit and secretion, and permeability), and through the luminal secretion of hormones that regulate microbial gene expression [3]. Earlier imaging studies have examined the associations of gut microbiota with brain structure and function in humans. For example, multimodal neuroimaging (including regional homogeneity and functional connectivity density, cerebral blood flow, gray matter volume, and fractional anisotropy) fusion biomarkers mediate the association between gut microbiota and cognition in healthy young adults [6]. Wang et al. revealed that gut microbiota alteration caused default mode network functional connectivity impairment by increasing systemic inflammation in end-stage renal disease [7]. Moreover, a recent longitudinal study demonstrated a significant influence of 4-week multi-strain probiotic administration on resting-state functional connectivity in healthy subjects [8], providing indirect support for the gut microbiota-brain function interaction. Nonetheless, there is a paucity of large sample and multimodal neuroimaging studies offering direct insight regarding the nature of the gut microbiota-brain relationship in healthy populations.

It is generally accepted that sex can influence the complexity and diversity of gut microbes and reciprocally the gut microbiota can affect sex steroid hormones [9]. Previous human studies have revealed considerable sex differences in the composition of gut microbiome [10]. In parallel, experimental animal research has corroborated the sexual dimorphism of gut microbiota observed in humans [11–13]. Conversely, there is empirical evidence that probiotics can regulate the levels of sex hormones by manipulating the intestinal microbiome in polycystic ovary syndrome patients [14]. In addition, extensive neuroimaging research has established the presence of sex differences in brain structure, perfusion, and function. With respect to brain structure, a prior large-scale study has reported higher gray matter volume, cortical surface area and white matter integrity in males, and higher cortical thickness and white matter tract complexity in females [15]. In regard to brain perfusion, females have higher global cerebral blood flow (CBF) than males [16]. As to brain function, males show stronger functional connectivity in unimodal sensorimotor cortex, while females exhibit stronger functional connectivity in default mode network [15]. Despite these findings, sex-dependent effects of gut microbiota on the brain are less clearly established, with only a more recent animal study suggesting that disruption of the gut microbiome affects hippocampal neurogenesis in a sex-dependent manner [17]. More broadly, as there has been a growing emphasis on the sex-specific influence of gut microbiota on mental disorders [18], elucidating such effects not only may aid in further understanding disease mechanisms, but also may have clinical implications for developing personalized medicine approaches.

In the current study, we collected fecal samples from a large sample of healthy young adults and utilized 16S rRNA gene amplicon sequencing technology to measure gut microbial diversity [19]. Structural MRI, arterial spin labeling (ASL), resting-state functional MRI (fMRI), and diffusion tensor imaging (DTI) were adopted to assess brain structure, perfusion, and function. Mounting evidence converges to support the concept that a conjoint analysis of multimodal imaging data would provide integrated information on complex underlying neurobiological features [20–22]. Additionally, the Go/No-Go task was employed to assess the ability of behavioral inhibition [23]. We focused our efforts on this cognitive domain because poor inhibitory control is thought to be a common symptom of a number of mental disorders, and sex-dependent associations between addiction-related behaviors and the microbiome were observed in some studies [24–26].

**Fig. 1:**
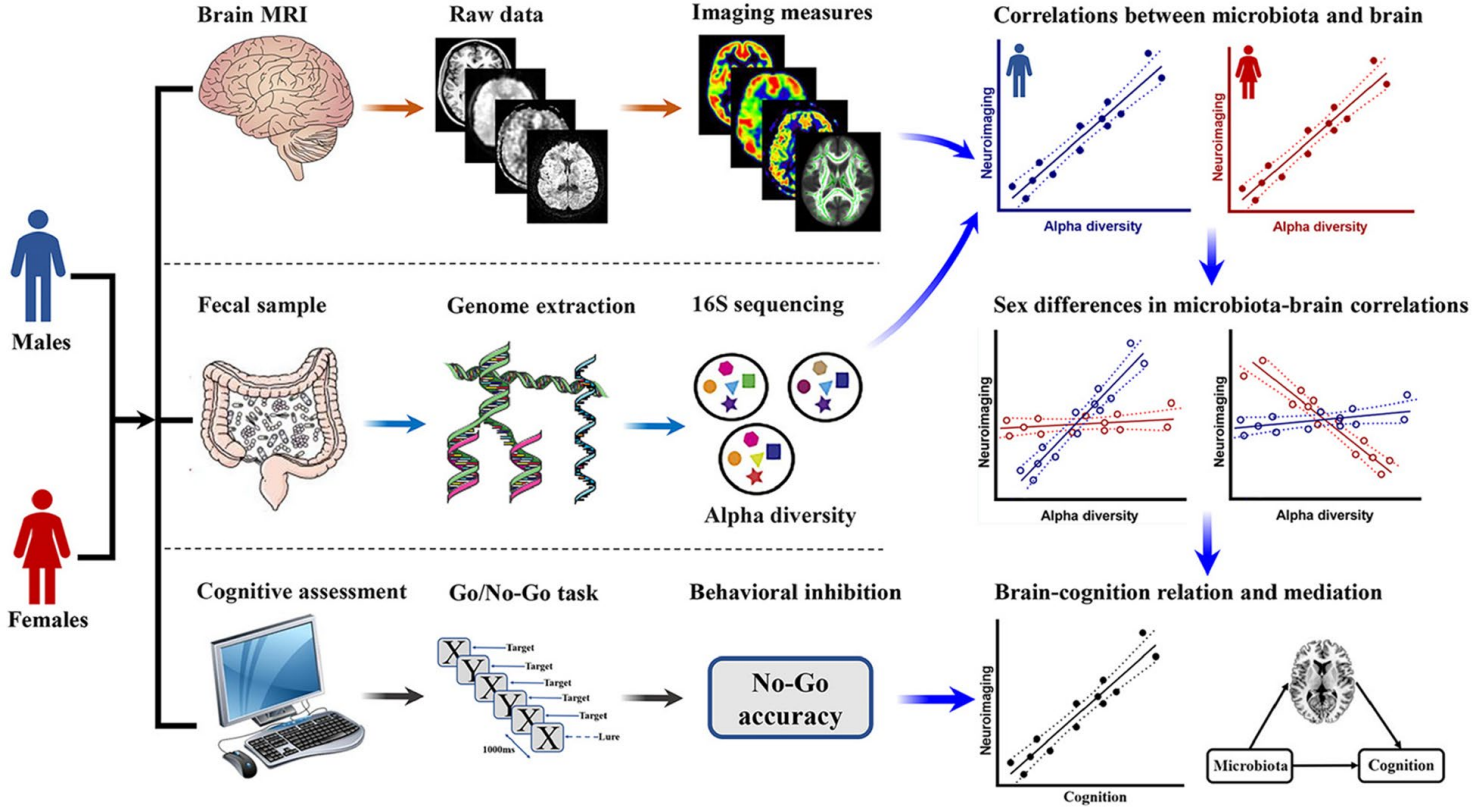
A flow chart of the research design. Abbreviation: MRI, magnetic resonance imaging

Based on this combined body of data, the first goal of this exploratory study was to examine the sex-dependent associations between gut microbial diversity and multimodal brain imaging measures. The second objective was to assess the sex-specific links between microbial diversity-associated brain imaging measures and behavioral inhibition. Finally, we sought to characterize the meditative role of the identified neuroimaging biomarkers in accounting for the sex-specific effects of gut microbial diversity on behavioral inhibition. A flow chart of the research design is shown in Fig. 1. Building on previous work, we hypothesized that there would be marked sexual dimorphism in the gut microbiota-brain-cognition relationships.

## Materials and methods

### Participants

A total of 157 healthy young adults were recruited by advertisement. All participants met the inclusion criteria of Chinese Han, right handedness, and within a restricted age range of 18–30 years. Exclusion criteria included neuropsychiatric or severe somatic disorder, a history of alcohol or drug abuse, regular smoker, menstruating females, current medication (e.g., antibiotics, sedative hypnotics or contraceptives) within a month, pregnancy, MRI contraindications, and a family history of psychiatric illness among first-degree relatives. The MINI-International Neuropsychiatric Interview (M.I.N.I.) and Alcohol Use Disorders Identification Test (AUDIT) were used in the process of excluding participants. This study was approved by the ethics committee of The First Affiliated Hospital of Anhui Medical University, all methods were carried out in accordance with relevant guidelines and regulations. Written informed consent was obtained from all participants after they had been given a complete description of the study.

### Go/No-Go task

The Go/No-Go task was conducted on a computer to assess the ability of behavioral inhibition using E-Prime 2.0 (http://www.pstnet.com/eprime.cfm) [23]. The primary variable of interest is the accuracy in “No-Go” conditions (Acc_No-Go) that reflects behavioral inhibition. The details are described in the online Supplemental methods.

### MRI data acquisition

High-resolution 3D T1-weighted structural images, perfusion imaging, resting-state blood-oxygen-level-dependent (BOLD) fMRI data, and DTI data were obtained using a 3.0-Tesla MR system (Discovery MR750w, General Electric, Milwaukee, WI, USA) with a 24-channel head coil. The details are described in the online Supplemental methods.

### Gray matter volume analysis

Voxel-based morphometry (VBM) analysis was performed using the CAT12 toolbox (http://www.neuro.uni-jena.de/cat) implemented in the Statistical Parametric Mapping software (SPM12, http://www.fil.ion.ucl.ac.uk/spm). First, all the structural T1-weighted images were corrected for bias-field inhomogeneities. Second, these images were segmented into gray matter, white matter and cerebrospinal fluid density maps using the “new-segment” approach [27]. Third, a diffeomorphic anatomical registration through the exponentiated Lie algebra (DARTEL) technique was used to generate a custom, study-specific template [28]. Fourth, each participant’s gray matter density image was warped to the customized template; then the resultant images were affine registered to the Montreal Neurological Institute (MNI) space and resampled to a voxel size of 1.5 mm × 1.5 mm × 1.5 mm. Fifth, the modulation was applied by multiplying the transformed gray matter density maps with the non-linear components of Jacobian determinants, which resulted in the normalized gray matter volume (GMV) maps representing the local native-space GMV after correcting the confounding effect of variance induced by individual whole-brain size. Finally, the resultant GMV images were smoothed with a 6 mm full-width at half maximum (FWHM) Gaussian kernel.

### Cerebral blood flow analysis

Three ASL difference images were calculated by subtracting the label images from the control images and then averaged. Next, CBF was quantified by applying a single-compartment model [29] to the mean ASL difference and proton-density-weighted reference images [30–32]. SPM12 software was used to normalize the CBF images into the MNI space using the following steps: (1) individual structural images were firstly co-registered with the CBF images; (2) the transformed structural images were segmented and normalized to the MNI space; and (3) the CBF image of each subject was written into the MNI space using the deformation parameter derived from the prior step and was resliced into a 2-mm cubic voxel. For the purpose of standardization, the CBF value of each voxel was divided by the global mean CBF value. Finally, the CBF images were smoothed with a 6 mm FWHM Gaussian kernel.

### Functional connectivity strength analysis

Resting-state BOLD data were preprocessed using SPM12 and Data Processing & Analysis for Brain Imaging (DPABI, http://rfmri.org/dpabi) [33]. The details are described in the online Supplemental methods.

Functional connectivity strength (FCS) is a graph theory measure that evaluates functional connectivity of each voxel with all other voxels across the whole gray matter [34–36]. Firstly, we computed Pearson’s correlation coefficients between the BOLD time courses of all pairs of voxels and obtained a whole gray matter functional connectivity matrix for each participant. For a given voxel, FCS was computed as the sum of positive functional connectivity above a threshold of 0.25 between that voxel and all other voxels within the whole gray matter. Then, we normalized the FCS value of each voxel by dividing it by the global mean FCS value. Finally, the FCS maps were smoothed with a 6 mm FWHM Gaussian kernel.

### White matter integrity analysis

For DTI data, standard processing steps were performed by using the FMRIB Software Library (FSL, www.fmrib.ox.ac.uk/fsl). First, eddy current distortion and head motion were corrected by registering the diffusion-weighted images to the first b0 image through the affine transformations. Second, the data were skull-stripped by using the FMRIB Brain Extraction Tool. Finally, diffusion parameters including fractional anisotropy (FA), axial diffusivity (AD), radial diffusivity (RD), and mean diffusivity (MD), were calculated by using the DTIFIT toolbox. Then, tract-based spatial statistics (TBSS) pipeline was conducted [37, 38]. Briefly, individual FA images were firstly non-linearly registered to the MNI space. After transformation into the MNI space, mean FA image was created and thinned to generate a mean FA skeleton. Then, each subject’s FA image was projected onto the skeleton via filling the mean FA skeleton with FA values from the nearest relevant tract center by searching perpendicular to the local skeleton structure for maximum FA value. Finally, the registration and projection information derived from the FA analysis was applied to the other diffusion parameters to project AD, RD, and MD images onto this common skeleton.

### Fecal samples collection and gut microbiota analysis

Microbial genome DNA was extracted from the fecal samples using a QIAamp DNA Stool Mini Kit (Qiagen Inc., Hilden, Germany). The V4 region of 16S ribosomal RNA (rRNA) gene was amplified. The qualified amplicon mixture was then sequenced on the MiSeq platform with the PE250 sequencing strategy. Alpha diversity was assessed using the species richness indices (Sobs, Chao, and Ace) and species diversity indices (Shannon and Simpson that reflect both species richness and species evenness) [39, 40], which were calculated by MOTHUR (v1.31.2) [41] and QIIME (v1.8.0) [42] at the operational taxonomic unit (OUT) level. The species accumulation curves were plotted in Supplementary Fig. S1, which indicated that the sampling amount was sufficient. The details are described in the online Supplemental methods.

### Statistical analysis

Demographic, cognitive, and gut microbial variables were compared between males and females using two sample *t*-tests in the SPSS 26.0 software (SPSS Inc., Chicago, IL, United States).

In the male and female groups separately, voxel-based partial correlation analyses between alpha diversity and brain imaging measures (GMV, CBF, and FCS) were performed using multiple regression analyses in the SPM12 software. For CBF analyses, age was included as a nuisance covariate, with total intracranial volume (TIV) and FD as additional covariates for GMV and FCS analyses respectively. Multiple comparisons were corrected using a cluster-level family-wise error (FWE) method, resulting in a cluster defining threshold of *p* = 0.001 and a corrected cluster significance of *p* < 0.05. For the TBSS analysis, non-parametric permutation testing (permutation number = 5000) and threshold-free cluster enhancement (TFCE) in the FSL software were used for statistical inference of the partial correlations between alpha diversity and diffusion parameters (AD, RD and MD) controlling for age. The FWE method was also used to correct for multiple comparisons with a corrected significance threshold of *p* < 0.05. In case of significant correlations identified for any brain regions in either males or females, these significant regions were defined as regions of interest (ROIs) and mean imaging values within these ROIs were extracted to further examine whether there were significant sex differences in the correlations. That is, ROI-based partial correlation coefficients between imaging measures and alpha diversity were transformed into Fisher’s Z scores and then compared between males and females [43]. Specifically, we compared correlation differences between groups using Fisher’s r to z transformation, so that z scores were compared and analyzed for statistical significance using z test statistics at a set alpha level (significance level). To estimate the effect sizes of sex, we also calculated Cohen’s *q* (no effect:* q* < 0.1, small effect: 0.1 < *q* < 0.3, intermediate effect: 0.3 < *q* < 0.5, large effect:* q* > 0.5) [44].

For brain imaging parameters showing correlations with gut microbial diversity, we further examined their associations with the ability of behavioral inhibition (Acc_No-Go) using partial correlation analyses. To test whether the association between variables was mediated by other variables, mediation analysis was performed using the PROCESS macro (http://www.processmacro.org/) [45]. In the mediation analysis model (Fig. 8A), all paths were reported as unstandardized ordinary least squares regression coefficients, namely, total effect of X on Y (c) = indirect effect of X on Y through M (a × b) + direct effect of X on Y (c’). The significance analysis was based on 5000 bootstrap realizations and a significant indirect effect is indicated when the bootstrap 95% confidence interval (CI) does not include zero. In this study, only variables that showed a significant correlation with others in the correlation analyses were considered independent (alpha diversity), dependent (cognition), or mediating variables (neuroimaging parameters) in the mediation analysis.

## Results

### Demographic, cognitive, and gut microbial characteristics

As shown in Table 1, males and females differed significantly in age (*t* = -2.84, *p* = 0.005), years of education (*t* = -3.60, *p* < 0.001), body mass index (BMI, *t* = 4.15, *p* < 0.001), TIV (*t* = 11.15, *p* < 0.001), and FD (*t* = 2.35, *p* = 0.020). Of note, Pearson’s correlation analyses revealed strong positive correlations (*r* > 0.95) between Sobs, Chao, and Ace indices as well as a strong negative correlation (*r* = -0.92) between Shannon and Simpson indices (Supplementary Table S1). Therefore, we reported the main results of Chao and Shannon analysis and provided the results of Sobs, Ace, and Simpson analysis in the Supplementary materials.

**Table 1:** Demographic and cognitive characteristics of the sample

Characteristics	Males	Females	*t* value	*p* value
Number of subjects	80	77	-	-
Age (years)	21.80 ± 2.39	22.87 ± 2.34	-2.84	0.005
Education (years)	15.26 ± 1.93	16.32 ± 1.77	-3.60	< 0.001
BMI (kg/m^2^)	22.42 ± 3.69	20.42 ± 2.18	4.15	< 0.001
TIV (cm^3^)	1569.97 ± 101.63	1393.49 ± 96.49	11.15	< 0.001
FD (mm)	0.13 ± 0.06	0.11 ± 0.03	2.35	0.020
Acc_No-Go	0.61 ± 0.19	0.58 ± 0.18	0.95	0.342

The data were presented as the mean ± SD

*Abbreviation*s: *SD* standard deviation, *BMI* body mass index, *TIV* total intracranial volume, *FD* frame-wise displacement, *Acc_No-Go* accuracy in “No-Go” conditions

### Sex differences in alpha diversity-GMV association

**Fig. 2:**
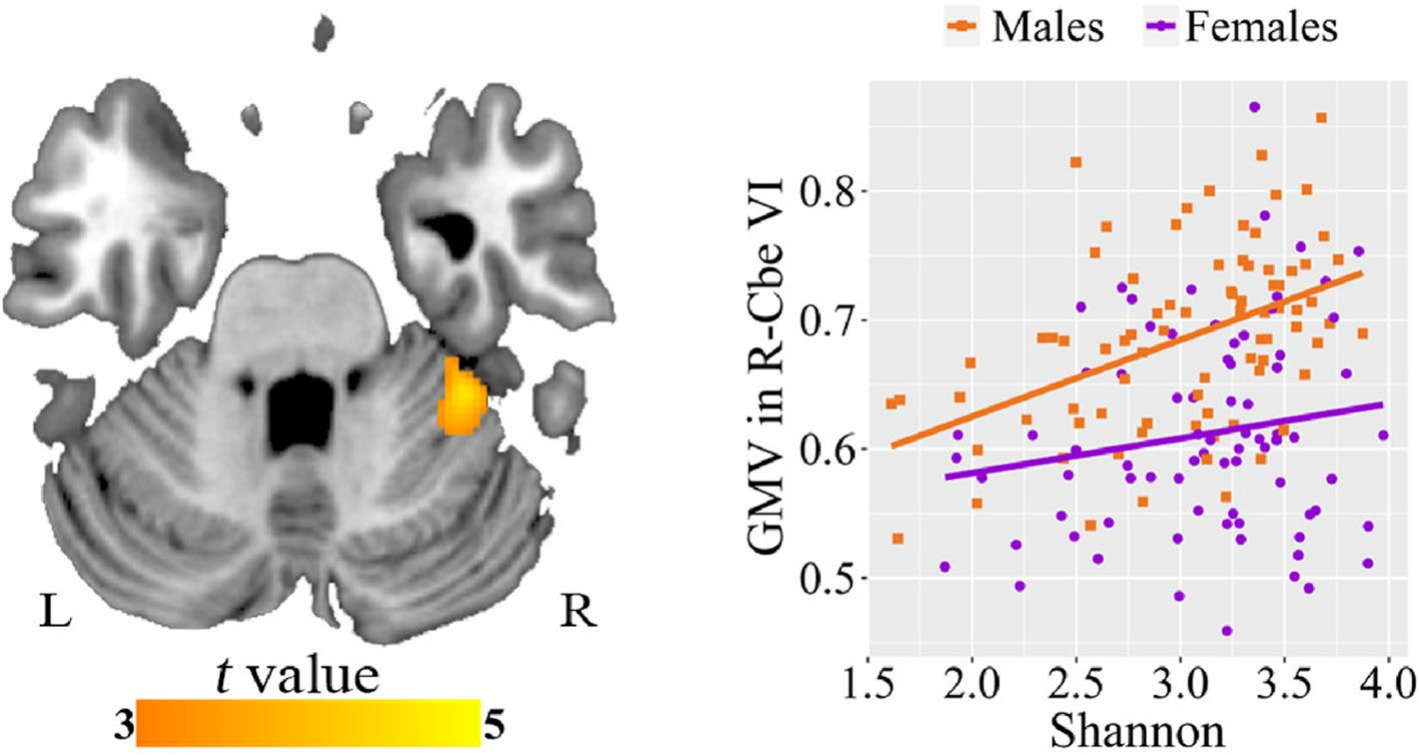
Sex differences in the associations between Shannon index and GMV. Voxel-based analysis reveals a positive correlation between Shannon index and GMV in R-Cbe VI in males. Scatter plots show the ROI-based correlations between Shannon index and GMV in R-Cbe VI in males and females, separately. Abbreviations: GMV, gray matter volume; Cbe VI, cerebellum VI; ROI, region of interest; R, right; L, left

After controlling for age and TIV, we found a significant positive correlation between Shannon index and GMV in the right cerebellum VI (R-Cbe VI, cluster size = 523 voxels, peak MNI coordinate x/y/z = 34.5/-37.5/-28.5, peak *t* = 4.56; *p* < 0.05, cluster-level FWE corrected) in males but not females (Fig. 2). ROI-based analysis further demonstrated a significant sex difference in the Shannon index-GMV association (Table 2). The difference was still significant after additional adjustment for education and BMI (Supplementary Table S2). The results of Simpson index analysis were similar to those of Shannon index analysis (Supplementary Fig. S2).

**Table 2:** Sex differences in the associations between Shannon index and brain imaging measures

Imaging measures	*r*_males_ (*p*)	*r*_females_ (*p*)	*Z* value of sex comparison in *r* (*p*)	Cohen’s *q*
GMV in R-Cbe VI	0.468 (< 0.001)	0.088 (0.452)	2.576 (0.010)	0.419
CBF in B-CAL	0.497 (< 0.001)	-0.227 (0.049)	4.769 (< 0.001)	0.776
CBF in L-SFG	-0.552 (< 0.001)	-0.069 (0.556)	-3.392 (< 0.001)	0.552
AD	-0.622 (< 0.001)	0.026 (0.825)	-4.633 (< 0.001)	0.754
RD	-0.533 (< 0.001)	0.014 (0.907)	-3.737 (< 0.001)	0.608
MD	-0.510 (< 0.001)	0.033 (0.778)	-3.660 (< 0.001)	0.596

*Abbreviations*: *GMV* gray matter volume, *CBF* cerebral blood flow, *AD* axial diffusivity, *RD* radial diffusivity, *MD* mean diffusivity, *Cbe VI* cerebellum VI, *CAL* calcarine sulcus, *SFG* superior frontal gyrus, *R* right, *B* bilateral, *L* left

### Sex differences in alpha diversity-CBF association

**Fig. 3:**
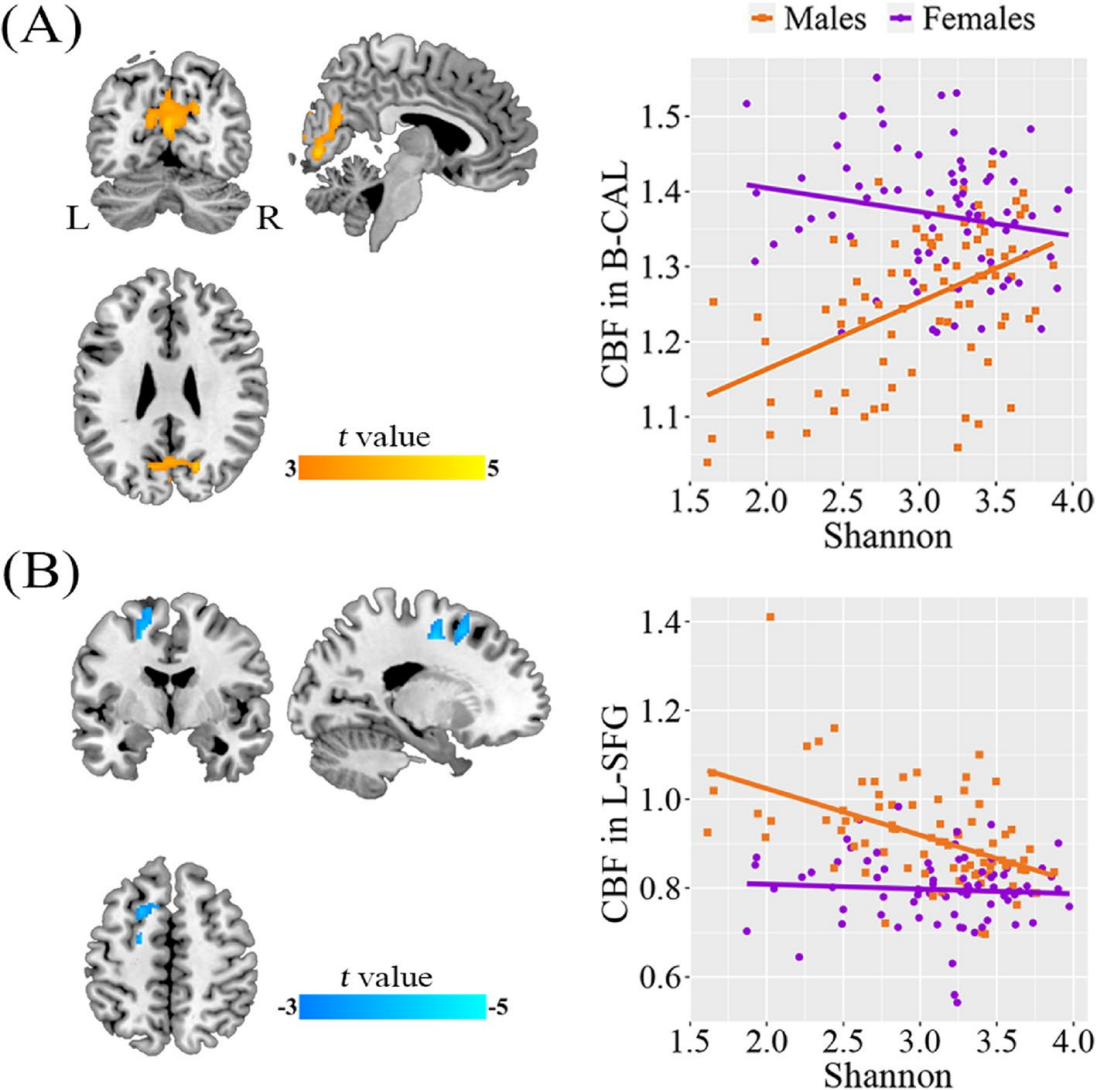
Sex differences in the associations between Shannon index and CBF. Voxel-based analysis reveals a positive correlation between Shannon index and CBF in B-CAL (**A**) and a negative correlation between Shannon index and CBF in L-SFG (**B**) in males. Scatter plots show the ROI-based correlations between Shannon index and CBF in B-CAL and L-SFG in males and females, separately. Abbreviations: CBF, cerebral blood flow; CAL, calcarine sulcus; SFG, superior frontal gyrus; ROI, region of interest; B, bilateral; R, right; L, left

After controlling for age, we found a positive correlation between Shannon index and CBF in the bilateral calcarine sulcus (B-CAL, cluster size = 781 voxels, peak MNI coordinate x/y/z = -2/-70/18, peak *t* = 4.52; *p* < 0.05, cluster-level FWE corrected) (Fig. 3A) and a negative correlation between Shannon index and CBF in the left superior frontal gyrus (L-SFG, cluster size = 268 voxels, peak MNI coordinate x/y/z = -20/-6/48, peak *t* = -4.41; *p* < 0.05, cluster-level FWE corrected) (Fig. 3B) in males but not females. ROI-based analysis further demonstrated significant sex differences in the Shannon index-CBF associations (Table 2). The differences were still significant after additional adjustment for education and BMI (Supplementary Table S2). The results of Simpson index analysis were similar to those of Shannon index analysis (Supplementary Fig. S3).

### Sex differences in alpha diversity-FCS association

**Fig. 4:**
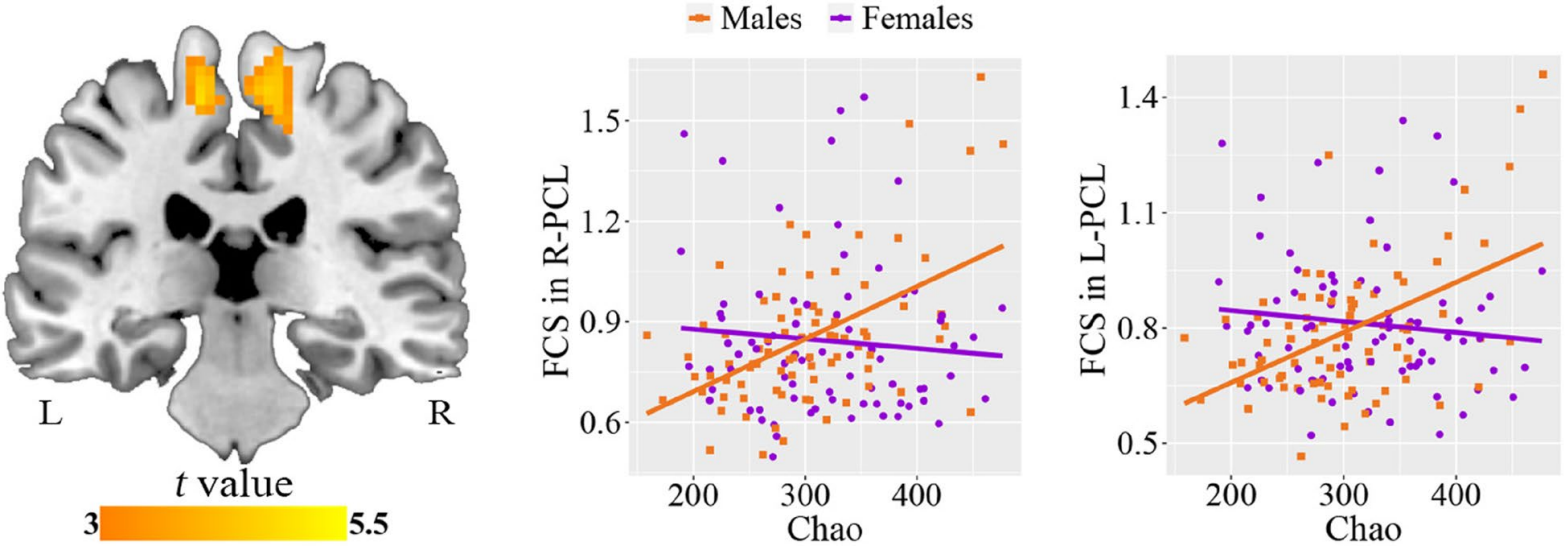
Sex differences in the associations between Chao index and FCS. Voxel-based analysis reveals positive correlations between Chao index and FCS in the bilateral PCL in males. Scatter plots show the ROI-based correlations between Chao index and FCS in the bilateral PCL in males and females, separately. Abbreviations: FCS, functional connectivity strength; PCL, paracentral lobule; ROI, region of interest; R, right; L, left

After controlling for age and FD, we found significant positive correlations between Chao index and FCS in the bilateral paracentral lobule (R-PCL: cluster size = 94 voxels, peak MNI coordinate x/y/z = 9/-33/60, peak *t* = 5.10; L-PCL: cluster size = 81 voxels, peak MNI coordinate x/y/z = -9/-21/63, peak *t* = 5.35; *p* < 0.05, cluster-level FWE corrected) in males but not females (Fig. 4). ROI-based analysis further demonstrated significant sex differences in the Chao index-FCS associations (Table 3). The differences remained significant after additional adjustment for education and BMI (Supplementary Table S3). The results of Sobs and Ace indices analysis were similar to those of Chao index analysis (Supplementary Fig. S4).

**Table 3:** Sex differences in the associations between Chao index and brain imaging measures

Imaging measures	*r*_males_ (*p*)	*r*_females_ (*p*)	*Z* value of sex comparison in *r* (*p*)	Cohen’s *q*
FCS in R-PCL	0.512 (< 0.001)	-0.082 (0.487)	3.978 (< 0.001)	0.648
FCS in L-PCL	0.508 (< 0.001)	-0.107 (0.362)	4.100 (< 0.001)	0.667
RD	-0.566 (< 0.001)	-0.040 (0.730)	-3.696 (< 0.001)	0.602
MD	-0.551 (< 0.001)	-0.013 (0.908)	-3.728 (< 0.001)	0.607

*Abbreviations*: *FCS* functional connectivity strength, *RD* radial diffusivity, *MD* mean diffusivity, *PCL* paracentral lobule, *R* right, *L* left

### Sex differences in alpha diversity-diffusion parameters association

**Fig. 5:**
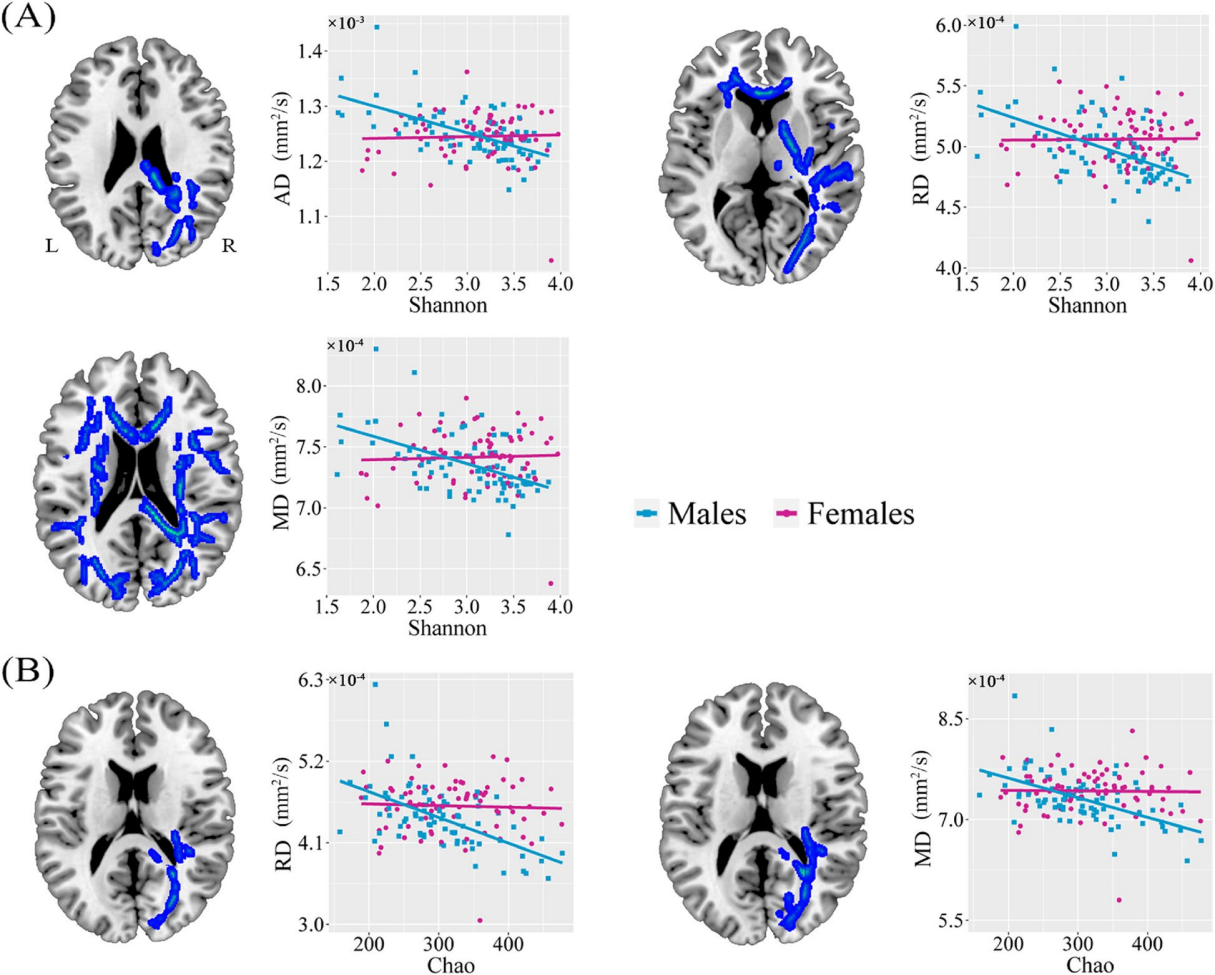
Sex differences in the associations of diffusion parameters with Shannon and Chao indices. Voxel-based analysis reveals negative correlations of diffusion parameters with Shannon (**A**) and Chao (**B**) indices across widespread white matter regions in males. Scatter plots show the ROI-based correlations of diffusion parameters with Shannon and Chao indices in males and females, separately. Abbreviations: AD, axial diffusivity; RD, radial diffusivity; MD, mean diffusivity; ROI, region of interest; R, right; L, left

After controlling for age, we found significant negative correlations between Shannon index and diffusion parameters (AD, RD, and MD) (Fig. 5A) and between Chao index and diffusion parameters (RD and MD) (Fig. 5B) across widespread white matter regions (*p* < 0.05, FWE corrected) in males but not females. ROI-based analysis further demonstrated significant sex differences in the Shannon index-diffusion parameters associations (Table 2) and Chao index-diffusion parameters associations (Table 3). These differences remained significant after additional adjustment for education and BMI (Supplementary Tables S2 and S3). While the results of Sobs index analysis were similar to those of Chao index analysis, the Ace index-diffusion parameters association was identified in a localized region and the Simpson index-diffusion parameters association was not significant (Supplementary Fig. S5).

### Gut microbiota-brain-cognition associations in males

**Fig. 6:**
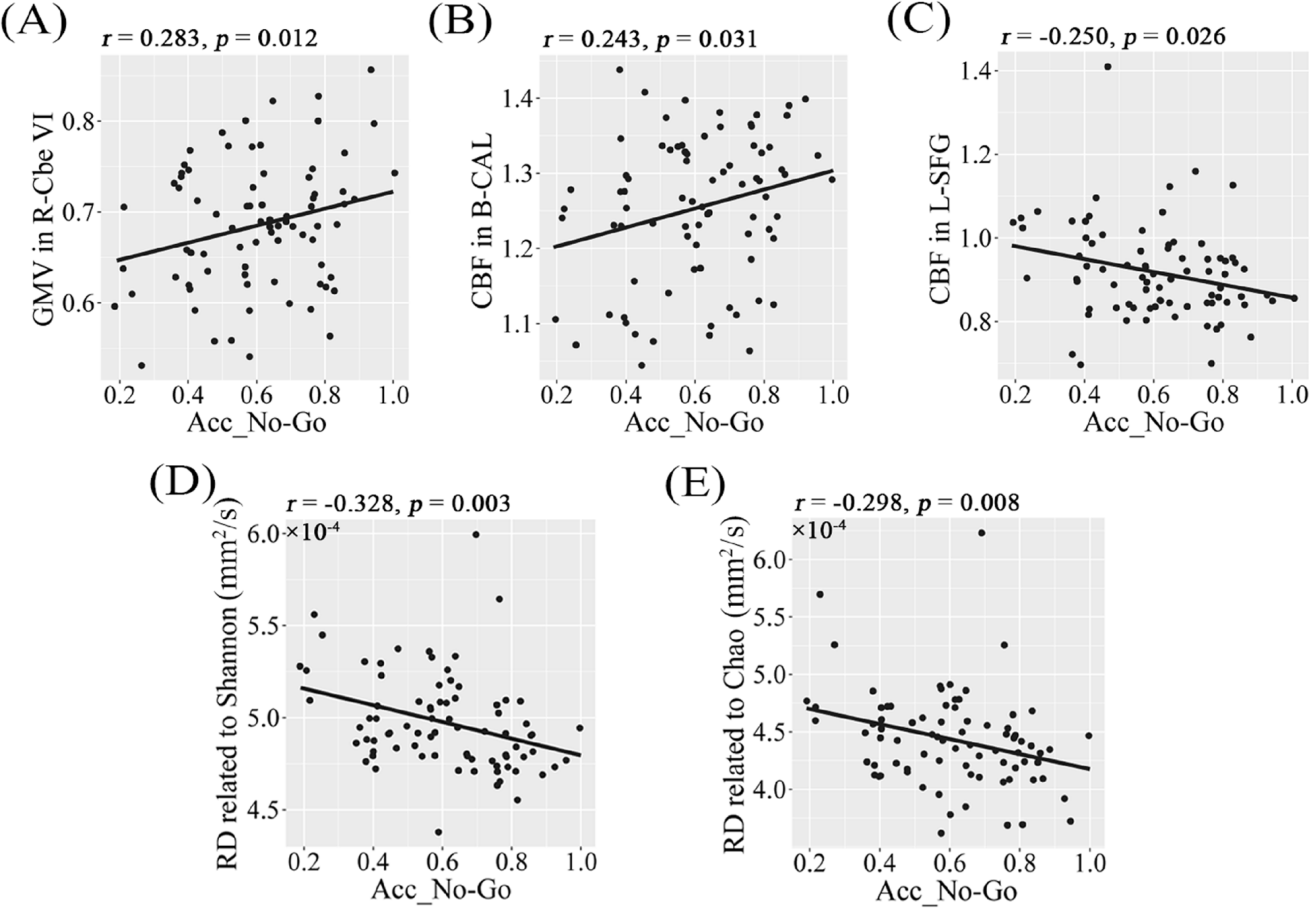
Scatter plots of correlations between alpha diversity-related neuroimaging parameters and Acc_No-Go in males. Abbreviations: Acc_No-Go, accuracy in “No-Go” conditions; GMV, gray matter volume; CBF, cerebral blood flow; RD, radial diffusivity; Cbe VI, cerebellum VI; CAL, calcarine sulcus; SFG, superior frontal gyrus; R, right; B, bilateral; L, left

**Fig. 7:**
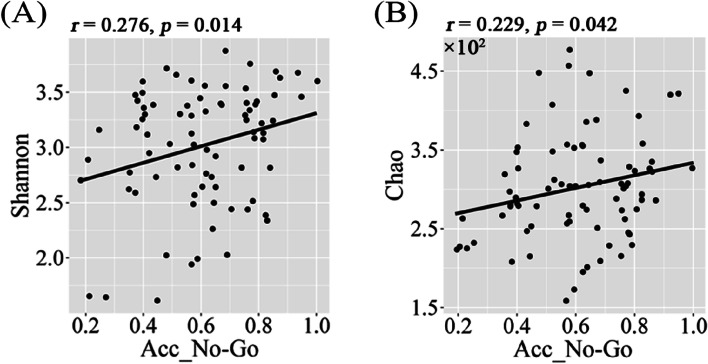
Scatter plots of correlations between alpha diversity and Acc_No-Go in males. Abbreviations: Acc_No-Go, accuracy in “No-Go” conditions

In light of significant correlations between gut microbial diversity and neuroimaging parameters in males rather than in females, we further assessed the relationships between microbial diversity-related imaging parameters and cognition in males. Results showed that Acc_No-Go was positively correlated with GMV in R-Cbe VI (Fig. 6A) and CBF in B-CAL (Fig. 6B), and was negatively correlated with CBF in L-SFG (Fig. 6C), RD related to Shannon index (Fig. 6D) and RD related to Chao index (Fig. 6E) in males. Critically, we also observed significant positive associations of Acc_No-Go with Shannon (Fig. 7A) and Chao (Fig. 7B) indices in males.

**Fig. 8:**
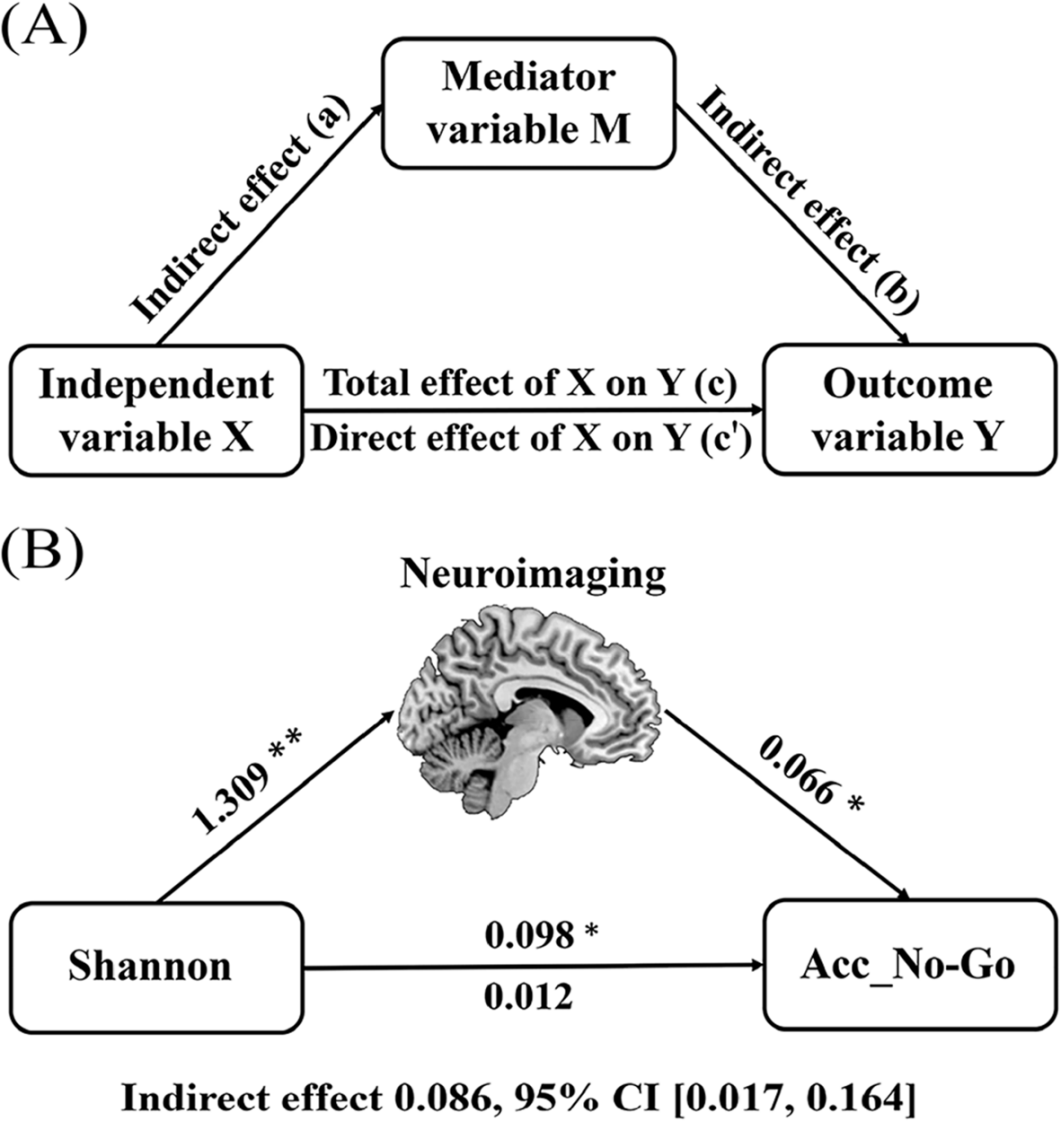
Conceptual diagram of mediation analysis. **A** Graphical representation of a mediation analysis model with one mediator. Total effect of X on Y (c) = indirect effect of X on Y through M (a × b) + direct effect of X on Y (c'). **B** The mediation analysis between Shannon index (X) and Acc_No-Go (Y), with the first neuroimaging component as the mediator (M). Path coefficients with *p* values (*p < 0.05 and **p < 0.01, respectively). Abbreviations: Acc_No-Go, accuracy in “No-Go” conditions

Considering these associations among alpha diversity, brain imaging parameters, and cognition in males, we adopted mediation analysis model to further characterize their relationship. To summarize individual differences in neuroimaging, a principal component analysis (PCA) was firstly performed to identify latent components underlying the four Shannon-related neuroimaging variables (GMV in R-Cbe VI, CBF in B-CAL, CBF in L-SFG, and RD). PCA is a technique to reduce the dimensionality of a data set composed of a large number of interrelated variables, while retaining as much of the variation present as possible in the data set [46]. Based on the Kaiser-Guttman criterion, components with an eigenvalue (EV) < 1.0 were removed. As a consequence, only the first neuroimaging component that accounted for 52% of the variance was retained and extracted for subsequent mediation analysis. Then, we found that the relationship between Shannon index and Acc_No-Go was significantly mediated by the first neuroimaging component (indirect effect = 0.086, SE = 0.037, 95% CI: 0.017, 0.164) in males (Fig. 8B). Moreover, the total effect of Shannon index on Acc_No-Go was significant (c = 0.098, SE = 0.039, *p* = 0.014) while the direct effect was insignificant (c’ = 0.012, SE = 0.053, *p* = 0.818), indicating a full mediation.

## Discussion

By using 16S amplicon sequencing to characterize gut microbiome diversity and multimodal MRI techniques to delineate brain properties, we conducted the first comprehensive analyses to investigate the effects of sex on the associations between the gut microbiota and the brain in a large sample of healthy young adults, and further explored the neural mechanism by which the gut microbiota influenced cognition in a sex-dependent way. Overall, we found specific gut microbiota-brain-cognition associations only in males, which is coherent with our hypothesis of sexual dimorphism in such relations. Specifically, higher gut microbial diversity was associated with higher GMV in Cbe VI, higher CBF in CAL yet lower CBF in SFG, higher FCS in PCL, and lower diffusivity in widespread white matter regions in males. However, these associations were absent in females. More importantly, these neuroimaging biomarkers were a significant mediator of the association between gut microbial diversity and behavioral inhibition in males. These findings suggest that sex may serve as a potential influential factor that needs to be taken into account when studying and interpreting the gut microbiota-brain-cognition relationships.

Past small sample studies have established the associations of gut microbiota diversity and structure with cerebellar structure and functional connectivity [47–49], highlighting the pivotal role of the cerebellum in the microbiota-gut-brain axis. Complementing and extending these previous findings, our large sample study further revealed that higher gut microbial diversity was associated with higher GMV in Cbe VI in males rather than females. Sex difference in this association may be attributed to sex differences in the composition [9], metabolites [50, 51], and immunity [52, 53] of the gut microbiota, which may render this cerebellar region particularly susceptible to gut microbial diversity in males. Cbe VI is considered a cerebellar functional hub that is of high connectivity to the cerebral cortex and subcortex [54]. Moreover, the cerebellum has been shown to play a role in inhibitory control [55]. The prior findings may explain the current observation of a link between higher GMV in Cbe VI and better ability of behavioral inhibition in males.

The gut microbiome can regulate neurovascular integrity, including CBF and blood–brain barrier (BBB) function [56, 57]. Impaired BBB function is also linked with CBF alterations [58]. Short-chain fatty acid (SCFA) produced by microorganisms can alter BBB permeability [59] and tight junctions of BBB [60, 61], which may result in CBF changes. Remarkably, the SCFA producing genera Prevotella, Ruminococcus and Roseburia are reported to depend on sex and hormonal status [9]. In addition, Sadler and colleagues found that SCFA supplementation in the drinking water of male mice significantly improved recovery of affected limb motor function, suggesting that microbiota-derived SCFA can modulate poststroke recovery in males [62]. All of these findings converge to support the notion that the gut microbiota might influence brain perfusion in a sex-dependent fashion, which is in agreement with our finding of associations of higher gut microbiome diversity with higher CBF in CAL yet lower CBF in SFG in males only. Furthermore, hyperperfusion in CAL and hypoperfusion in SFG were both found to relate to better Go/No-Go task performance, which is consistent with previous studies emphasizing the importance of these brain regions in cognitive functions [63, 64]. In combination, these findings have led to some speculation that the visual and executive control networks might be implicated in inhibitory control synergistically.

Some studies have attempted to assess the relationships between brain functional connectivity and gut microbiota. For example, Gao et al. reported that gut alpha diversity was associated with functional connectivity between the amygdala and thalamus, between the anterior cingulate cortex and anterior insula, and between the supplementary motor area and inferior parietal lobule in infants [65]. Curtis et al. showed that insular resting-state functional connectivity was related to gut microbiota diversity [48]. Simpson and colleagues found that microbiome depletion by antibiotics resulted in altered functional connectivity among brain regions [66]. Moreover, a longitudinal fMRI study revealed that consumption of a fermented milk product with probiotic for 4 weeks was associated with changes in midbrain functional connectivity in healthy women, partially in favor of the association between gut microbiota changes and functional connectivity alterations [67]. However, sex effects have not been considered in these previous studies. In this study, we observed that higher gut microbial diversity was associated with higher FCS in PCL in males but not females, yielding direct and complementary insight into the sex-specific mechanisms by which the gut microbiota exert effects on the sensorimotor network functional connectivity.

DTI is the most commonly used MRI technique to evaluate brain white matter integrity [38]. Taking advantage of DTI, extensive animal and human research has established the presence of links between gut microbiota and brain microstructure in healthy and clinical conditions [68–71]. In line with these pilot studies, we found that the higher gut microbiota diversity was associated with lower water diffusion across widespread white matter regions in males but not females. While the underlying cellular mechanisms are unknown, strongly affected AD, RD, and MD suggest that axonal myelination, fiber coherence, axonal diameter, packing density, and permeability levels may all contribute to these white matter integrity changes [72–74]. Notably, higher RD was found to correlate with lower accuracy in “No-Go” conditions, implying that a disruption of the myelin sheath rather than pure axonal damage may underlie worsen behavioral inhibition.

The reasons for the sex differences in gut microbiota-brain-cognition relationships are unknown and are likely to be multifactorial. Among them, sex hormones may contribute most to the observed dimorphism. On one hand, the gut microbiota and the brain may be differentially affected by estrogen and androgen [9, 75–77]. On the other hand, it is evident that estrogen and progesterone fluctuation over the menstrual cycle is related to brain structural and functional changes in females [78]. Thus, substantial changes in estrogen and progesterone levels over the menstrual cycle may explain the lack of significant gut microbiota-brain associations in females. In addition, we cannot rule out the possibility that the higher levels of testosterone and its stable concentration across the life span in males [79] may also contribute to the sex dimorphism.

Our mediation analysis suggests that the relationship between gut microbial diversity and behavioral inhibition ability can be fully mediated by the neuroimaging biomarkers (including GMV, CBF, and diffusion parameters) in males. Theoretically, it provides preliminary evidence that the effects of gut microbiome on cognition appear to have a sex-dependent neuroanatomical basis. Moreover, this finding is of high clinical and translational importance, which may expose the gut microbiota as a biomarker-driven and sex-sensitive intervention target for mental disorders with abnormal behavioral inhibition. This may ultimately inform a novel conceptualization of how to treatment these disorders via the regulation of gut microbiota in a personalized manner.

Several limitations should be noted in our research. First, the cross-sectional design limits our ability to make causal inferences. Future prospective longitudinal studies are needed to resolve causality of the complex gut microbiota-brain-cognition relationship. Second, we only focused on the correlations between gut microbiome diversity and the brain. Further investigations are required to determine whether and how certain bacteria are linked to brain structure and function in a sex-dependent manner. Third, since this study population was selected from a group of educated volunteers with an age range of 18–30 years, these findings might not be representative of the general population. Future studies may benefit from enrolling a sample of subjects with broader age and educational ranges. Finally, multiple testing corrections were not performed for multimodal brain imaging measures. However, the voxel-based statistical test for each imaging measure was corrected for multiple comparisons with appropriate methods. Type II error control is equally important because our analyses are exploratory in nature and important for future hypotheses generation.

In conclusion, this is to our knowledge the first multimodal MRI study demonstrating sex differences in the correlations between gut microbiota and brain structure, perfusion, and function in a large cohort of healthy young adults. In accordance with our expectations, we found specific gut microbiota-brain-cognition associations in males rather than females. More generally, these findings may contribute to groundwork for future individualized, biomarker-driven and sex-sensitive interventions of mental disorders by targeting the microbiota-gut-brain axis.

## Supplementary Information

**Additional file 1:** **Figure S1. **Species accumulation curves. **Figure S2. **Sex differences in the associations between Simpson index and GMV. **Figure S3. **Sex differences in the associations between Simpson index and CBF. **Figure S4. **Sex differences in the associations of FCS with Sobs and Ace indices. **Figure S5. **Sex differences in the associations of diffusion parameters with Sobs and Ace indices. **Table S1.** Correlations between alpha diversity indices. **Table S2.** Sex differences in the associations between Shannon index and brain imaging measures after additional adjustment for education and BMI. **Table S3. **Sex differences in the associations between Chao index and brain imaging measures after additional adjustment for education and BMI. 

## Data Availability

The raw DNA sequence data were deposited in the National Center for NCBI Sequence Read Archive (https://www.ncbi.nlm.nih.gov/bioproject/PRJNA793133).

## Abbreviations

AD: Axial diffusivity
Acc_No-Go: Accuracy in “No-Go” conditions
ASL: Arterial spin labeling
BMI: Body mass index
BBB: Blood–brain barrier
Cbe VI: Cerebellum VI
CBF: Cerebral blood flow
CAL: Calcarine sulcus
DTI: Diffusion tensor imaging
FCS: Functional connectivity strength
FWHM: Full-width at half maximum
FA: Fractional anisotropy
GMV: Gray matter volume
L: Left, MRI: magnetic resonance imaging
MD: Mean diffusivity
OUT: Operational taxonomic unit
PCL: Paracentral lobule
ROI: Region of interest
RD: Radial diffusivity
SFG: Superior frontal gyrus
SCFA: Short-chain fatty acid
TIV: Total intracranial volume
