# Supplementary material for: Sex-dependent gut microbiota-brain-cognition associations: a multimodal MRI study
Source: BMC Neurol. 2023 Apr 27;23:169. doi: 10.1186/s12883-023-03217-3 (PMC10134644; doi:10.1186/s12883-023-03217-3)
Supplement: Supplementary file 1 — Additional file 1: Figure S1. Species accumulation curves. Figure S2. Sex differences in the associations between Simpson index and GMV. Figure S3. Sex differences in the associations between Simpson index and CBF. Figure S4. Sex differences in the associations of FCS with Sobs and Ace indices. Figure S5. Sex differences in the associations of diffusion parameters with Sobs and Ace indices. Table S1. Correlations between alpha diversity indices. Table S2. Sex differences in the associations between Shannon index and brain imaging measures after additional adjustment for education and BMI. Table S3. Sex differences in the associations between Chao index and brain imaging measures after additional adjustment for education and BMI. [file 12883_2023_3217_MOESM1_ESM.doc]

**Supplementary material**

**Supplementary methods**

**Go/No-Go task**

The Go/No-Go task was conducted on a computer to assess the ability of behavioral inhibition using E-Prime 2.0 (<http://www.pstnet.com/eprime.cfm>) [1]. During the task, the letter X or Y was presented at a frequency of 1 Hz on the screen. In “Go” conditions, the current letter is different from the previous one and participants should respond quickly by pressing the button within 900 ms. In “No-Go” conditions (10% of all trials), the current letter is the same as the previous one and participants cannot press the button; if one presses the button, it would be counted as an error. The Go/No-Go task consisted of a practice test and a formal test. There were 20 trials (15 “Go” trials and 5 “No-Go” trials) in the practice test. If a participant responds correctly in 3 “No-Go” trials, he or she can shift to the formal test; otherwise, the participant needs to restart the practice test. The formal test was divided into two groups with 210 trials in each group and 30 s break between the two groups. It took about 12 min for the Go/No-Go task. The primary variable of interest is the accuracy in “No-Go” conditions (Acc_No-Go) that reflects behavioral inhibition.

**MRI data acquisition**

MRI scans were obtained using a 3.0-Tesla MR system (Discovery MR750w, General Electric, Milwaukee, WI, USA) with a 24-channel head coil. Earplugs were used to reduce scanner noise, and tight but comfortable foam padding was used to minimize head motion. High-resolution 3D T1-weighted structural images were acquired by employing a brain volume (BRAVO) sequence with the following parameters: repetition time (TR) = 8.5 ms; echo time (TE) = 3.2 ms; inversion time (TI) = 450 ms; flip angle = 12°; field of view (FOV) = 256 mm × 256 mm; matrix size = 256 × 256; slice thickness = 1 mm, no gap; 188 sagittal slices. The perfusion imaging was performed using a pseudo-continuous ASL sequence with a 3D fast spin-echo acquisition and background suppression (TR = 5070 ms, TE = 11.5 ms; post-label delay = 2025 ms; spiral in readout of eight arms with 512 sample points; flip angle = 111°; FOV = 240 mm × 240 mm; reconstruction matrix = 128 × 128; slice thickness = 3 mm, no gap; 50 axial slices; number of excitation = 3). The label and control whole-brain image volumes required 8 TRs, respectively. A total of three pairs of label and control volumes were acquired. Resting-state blood-oxygen-level-dependent (BOLD) fMRI data were acquired using a gradient-echo single-shot echo planar imaging (GRE-SS-EPI) sequence with the following parameters: TR = 2000 ms; TE = 30 ms; flip angle = 90°; FOV = 220 mm × 220 mm; matrix size = 64 × 64; slice thickness = 3 mm, slice gap = 1 mm; 35 interleaved axial slices; 185 volumes. DTI data were acquired using a spin-echo single-shot echo planar imaging (SE-SS-EPI) sequence with the following parameters: TR = 10000 ms; TE = 74 ms; flip angle = 90°; FOV = 256 mm × 256 mm; matrix = 128 × 128; slice thickness = 3 mm without gap; 50 axial slices; 64 diffusion gradient directions (b = 1000 s/mm^2^) plus five b = 0 reference images. All images were visually inspected to ensure that only images without visible artifacts were included in subsequent analyses.

**Resting-state blood-oxygen-level-dependent (BOLD) fMRI data preprocessing**

Resting-state BOLD data were preprocessed using SPM12 and Data Processing & Analysis for Brain Imaging (DPABI, [http://rfmri.org/dpabi](../../../../C:%5CUsers%5C123%5CDesktop%5C投稿%5CGut%5C%20http:%5Crfmri.org%5Cdpabi)) [2]. The first 10 volumes for each participant were discarded to allow the signal to reach equilibrium and the participants to adapt to the scanning noise. The remaining volumes were corrected for the acquisition time delay between slices. Then, realignment was performed to correct the motion between time points. Head motion parameters were computed by estimating the translation in each direction and the angular rotation on each axis for each volume. All participants’ BOLD data were within the defined motion thresholds (i.e., translational or rotational motion parameters less than 2 mm or 2°). We also calculated frame-wise displacement (FD), which indexes the volume-to-volume changes in head position. Several nuisance covariates (the linear drift, the estimated motion parameters based on the Friston-24 model, the spike volumes with FD > 0.5, the white matter signal, and the cerebrospinal fluid signal) were regressed out from the data. The datasets were then band-pass filtered using a frequency range of 0.01 to 0.1 Hz. In the normalization step, individual structural images were firstly co-registered with the mean functional image; then the transformed structural images were segmented and normalized to the MNI space using a high-level nonlinear warping algorithm, that is, the DARTEL technique. Finally, each filtered functional volume was spatially normalized to MNI space using the deformation parameters estimated during the above step and resampled into a 3-mm cubic voxel.

**Fecal samples collection and gut microbiota analysis**

Fecal samples were collected in sterilized tubes and stored immediately in a -80 ℃ freezer within 1 day before or after MRI examination. Microbial genome DNA was extracted from the fecal samples using a QIAamp DNA Stool Mini Kit (Qiagen Inc., Hilden, Germany). To construct the Polymerase Chain Reaction (PCR)-based 16S rDNA amplicon library for sequencing, PCR enrichment of the V4 hypervariable region of 16S rDNA was performed with the forward primer 515F (5’-GTGCCAGCMGCCGCGGTAA-3’) and reverse primer 806R (5’-GGACTACHVGGGTWTCTAAT-3’). The qualified amplicon mixture was then sequenced on the MiSeq platform with the PE250 sequencing strategy. Before the 16S rDNA data analysis, raw reads were filtered to remove adaptors and low-quality and ambiguous bases, and then paired-end reads were added to tags by the Fast Length Adjustment of Short reads program (FLASH, v1.2.11) [3]. The tags were clustered into operational taxonomic units (OTUs) with a cutoff value of 97% using UPARSE software (v9.1.13) [4] and chimera sequences were compared with the Gold database using UCHIME (v4.2.40) [5] to detect. Then, the representative sequence from each OTU cluster was obtained. These OTU representative sequences were taxonomically classified using Ribosomal Database Project (RDP) Classifier (v.2.2) [6] with a minimum confidence threshold of 0.8, and the training database was the Greengene Database (v201305) [7]. The USEARCH_global [8] was used to compare all tags back to OTU to get the OTU abundance statistics table of each sample. Alpha diversity was assessed using the species richness indices (Sobs, Chao, and Ace) and species diversity indices (Shannon and Simpson that reflect both species richness and species evenness) [9, 10], which were calculated by MOTHUR (v1.31.2) [11] and QIIME (v1.8.0) [12] at the OTU level. The species accumulation curves were plotted in supplementary Fig. S1, which indicated that the sampling amount was sufficient.

**References**

1. Kaufman JN, Ross TJ, Stein EA, and G. H, Cingulate hypoactivity in cocaine users during a GO-NOGO task as revealed by event-related functional magnetic resonance imaging, J Neurosci. 2003;23:7839-43.

2. Yan, C.G., X.D. Wang, X.N. Zuo, and Y.F. Zang, DPABI: Data Processing & Analysis for (Resting-State) Brain Imaging, Neuroinformatics. 2016;14:339-51.

3. Magoc, T. and S.L. Salzberg, FLASH: fast length adjustment of short reads to improve genome assemblies, Bioinformatics. 2011;27:2957-63.

4. Edgar, R.C., UPARSE: highly accurate OTU sequences from microbial amplicon reads, Nat Methods. 2013;10:996-8.

5. Edgar, R.C., B.J. Haas, J.C. Clemente, C. Quince, and R. Knight, UCHIME improves sensitivity and speed of chimera detection, Bioinformatics. 2011;27:2194-200.

6. Wang, Q., G.M. Garrity, J.M. Tiedje, and J.R. Cole, Naive Bayesian classifier for rapid assignment of rRNA sequences into the new bacterial taxonomy, Appl Environ Microbiol. 2007;73:5261-7.

7. DeSantis, T.Z., P. Hugenholtz, N. Larsen, M. Rojas, E.L. Brodie, K. Keller, et al., Greengenes, a chimera-checked 16S rRNA gene database and workbench compatible with ARB, Appl Environ Microbiol. 2006;72:5069-72.

8. Edgar, R.C., Search and clustering orders of magnitude faster than BLAST, Bioinformatics. 2010;26:2460-1.

9. Faith, D.P., Conservation evaluation and phylogenetic diversity. Biological Conservation, Biological Conservation. 1992;61:1-10.

10. Keylock, C.J., Simpson diversity and the Shannon–Wiener index as special cases of a generalized entropy, Oikos. 2005;109:203-207.

11. Schloss, P.D., S.L. Westcott, T. Ryabin, J.R. Hall, M. Hartmann, E.B. Hollister, et al., Introducing mothur: open-source, platform-independent, community-supported software for describing and comparing microbial communities, Appl Environ Microbiol. 2009;75:7537-41.

12. J Gregory Caporaso, Justin Kuczynski, Jesse Stombaugh, Kyle Bittinger, Frederic D Bushman, Elizabeth K Costello, et al., QIIME allows analysis of high-throughput community sequencing data. Nat Methods, Nat Methods. 2010;7:335-336.

**Supplementary figures and tables**


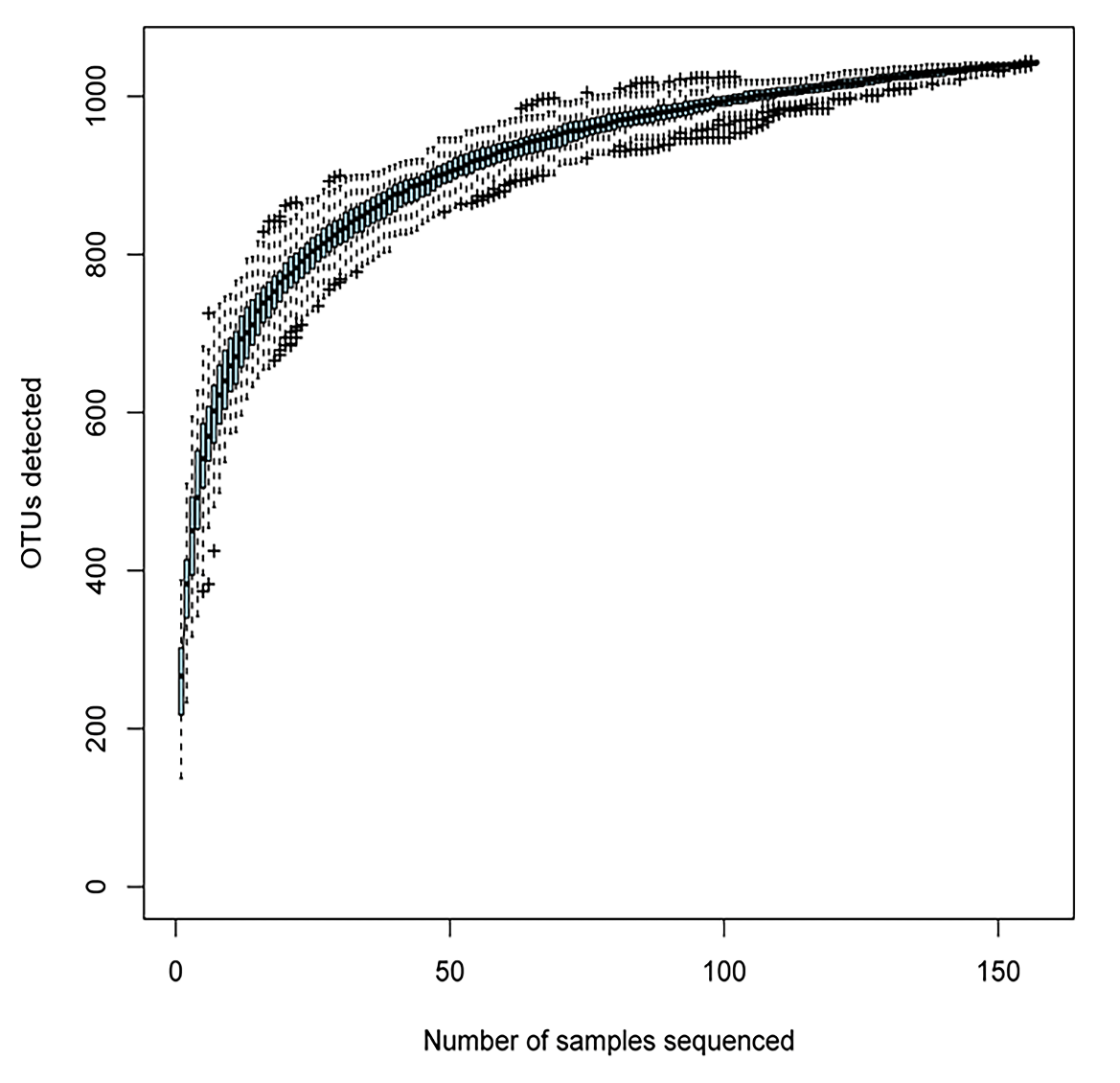


**Figure S1.** Species accumulation curves. The abscissa represents the number of samples, and the ordinate represents the number of OTUs (the number of detected species). Species accumulation curves reflect the influence of the number of samples on species diversity. The end of the curve tends to be flat, indicating that the sampling amount is sufficient. Abbreviations: OTU, operational taxonomic unit.


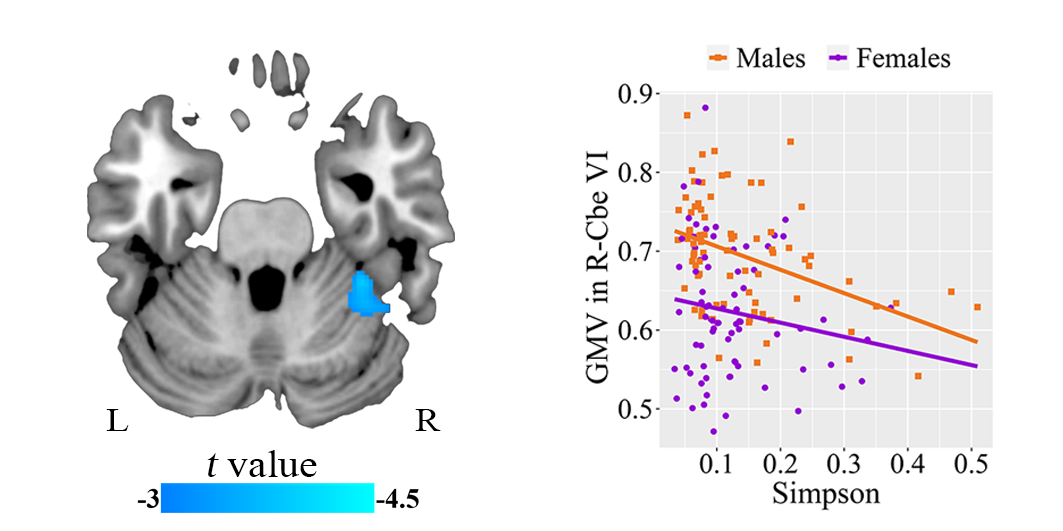


**Figure S2.** Sex differences in the associations between Simpson index and GMV. Voxel-based analysis reveals a negative correlation between Simpson index and GMV in R-Cbe Ⅵ in males. Scatter plots show the ROI-based correlations between Simpson index and GMV in R-Cbe Ⅵ in males and females, separately. Abbreviations: GMV, gray matter volume; Cbe Ⅵ, cerebellum Ⅵ; ROI, region of interest; R, right; L, left.


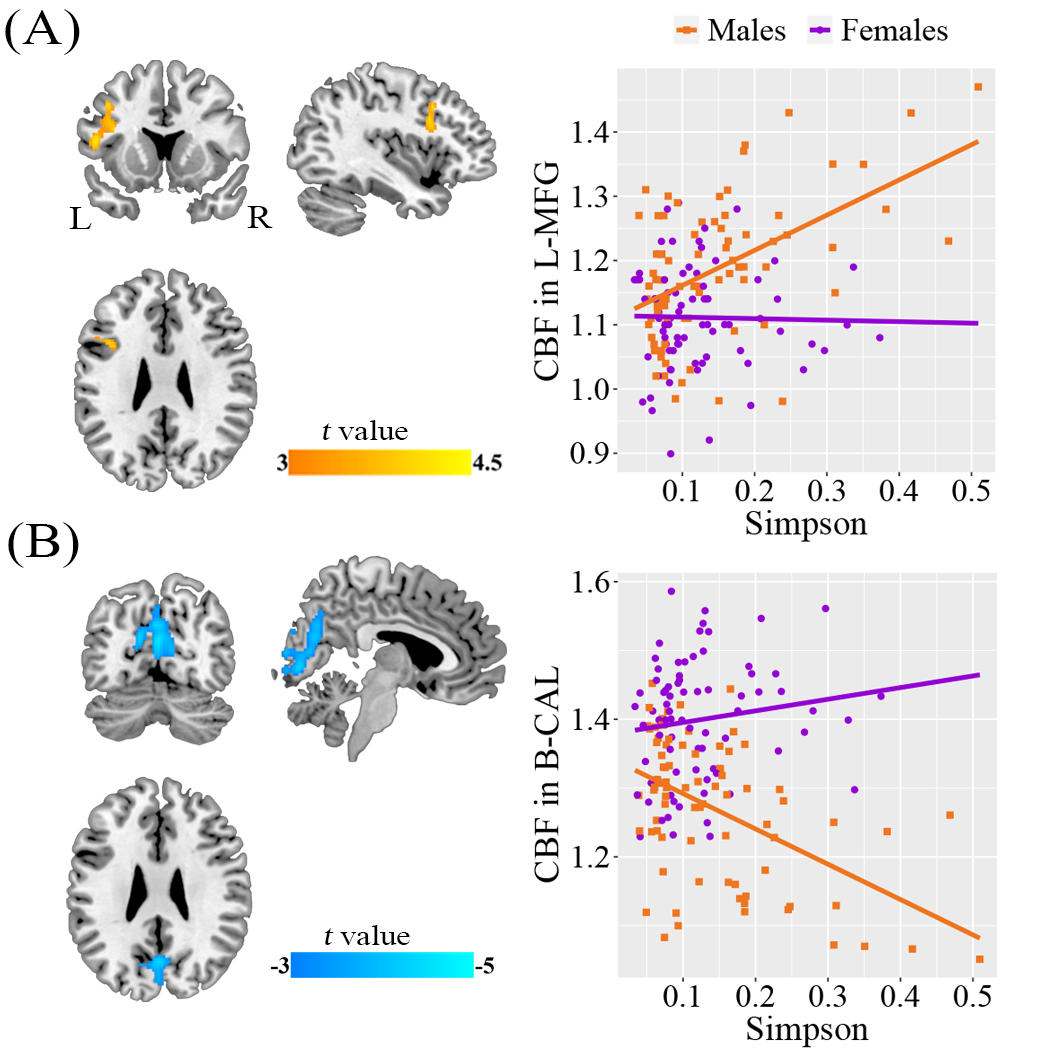


**Figure S3.** Sex differences in the associations between Simpson index and CBF. Voxel-based analysis reveals a positive correlation between Simpson index and CBF in L-MFG (A) and a negative correlation between Simpson index and CBF in B-CAL (B) in males. Scatter plots show the ROI-based correlations between Simpson index and CBF in L-MFG and B-CAL in males and females, separately. Abbreviations: CBF, cerebral blood flow; MFG, middle frontal gyrus; CAL, calcarine sulcus; ROI, region of interest; B, bilateral; R, right; L, left.


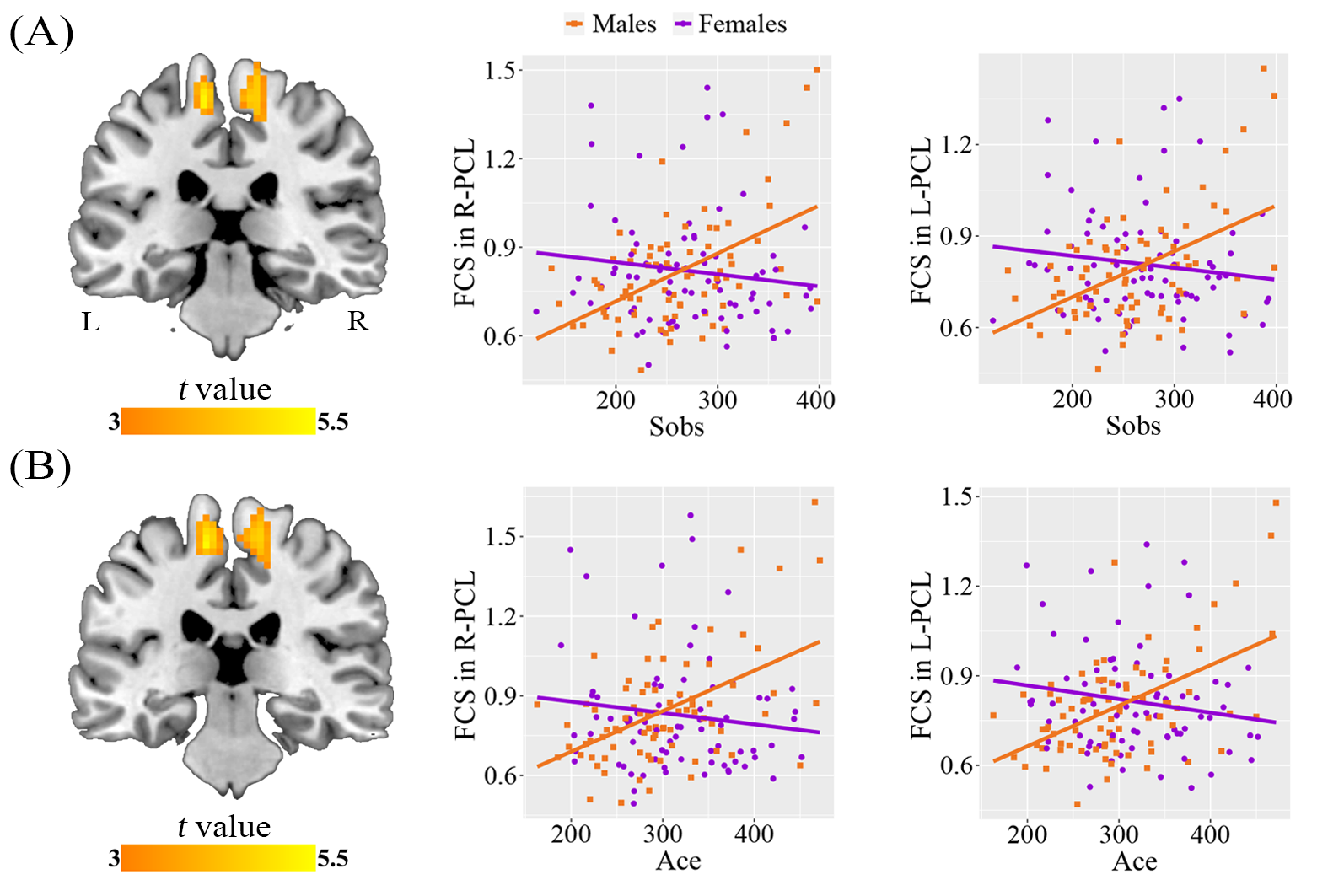


**Figure S4.** Sex differences in the associations of FCS with Sobs and Ace indices. Voxel-based analysis reveals positive correlations of FCS in the bilateral PCL with Sobs (A) and Ace (B) indices in males. Scatter plots show the ROI-based correlations of FCS in the bilateral PCL with Sobs and Ace indices in males and females, separately. Abbreviations: FCS, functional connectivity strength; PCL, paracentral lobule; ROI, region of interest; R, right; L, left.


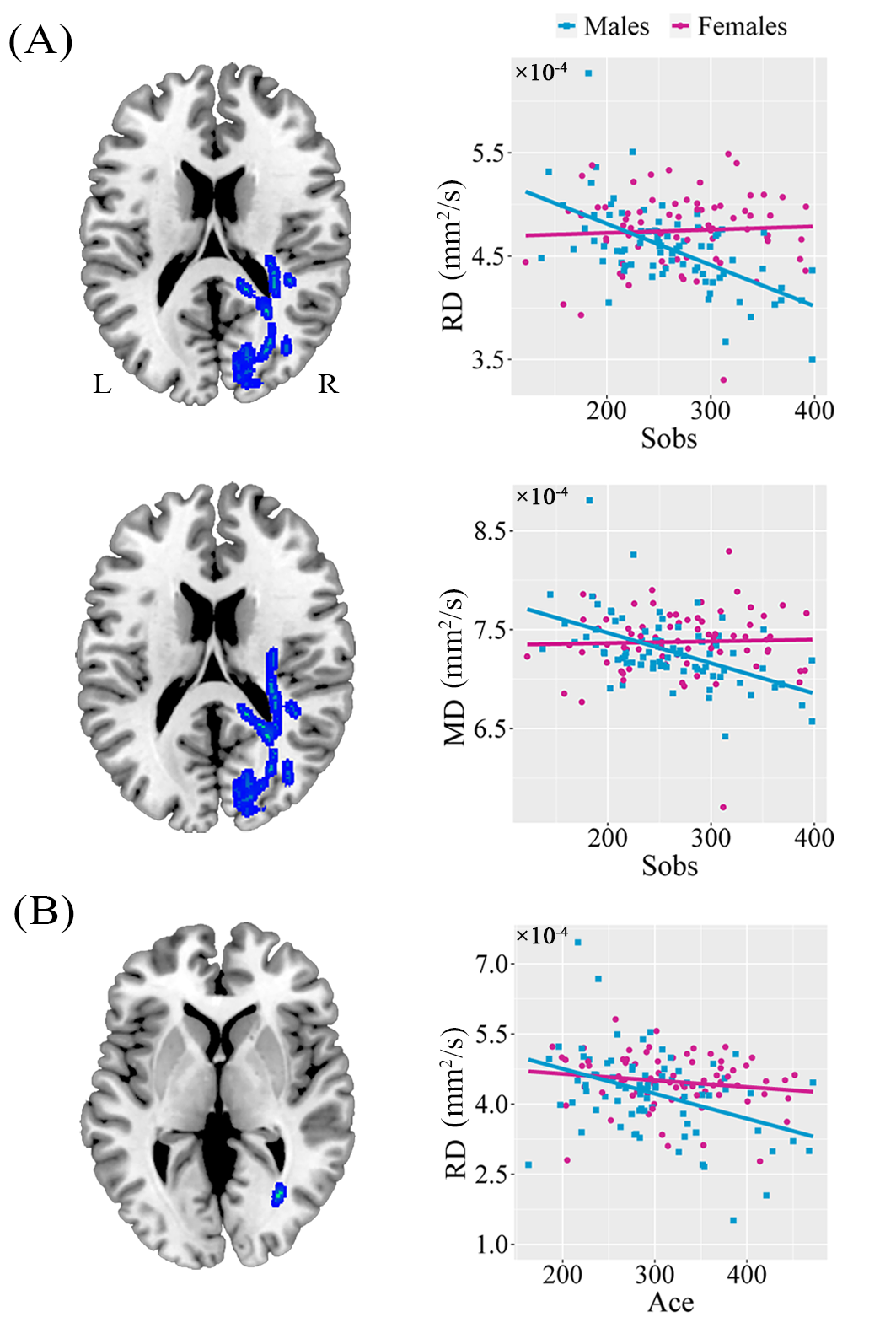


**Figure S5.** Sex differences in the associations of diffusion parameters with Sobs and Ace indices. Voxel-based analysis reveals negative correlations of diffusion parameters with Sobs (A) and Ace (B) indices in males. Scatter plots show the ROI-based correlations of diffusion parameters with Sobs and Ace indices in males and females, separately. Abbreviations: RD, radial diffusivity; MD, mean diffusivity; ROI, region of interest; R, right; L, left.

**Table S1.** Correlations between alpha diversity indices

|  | Sobs | Chao | Ace | Shannon | Simpson |
| --- | --- | --- | --- | --- | --- |
| Sobs | - | 0.966 | 0.957 | 0.488 | -0.284 |
| Chao | - | - | 0.980 | 0.423 | -0.240 |
| Ace | - | - | - | 0.399 | -0.226 |
| Shannon | - | - | - | - | -0.921 |
| Simpson | - | - | - | - | - |

**Table S2.** Sex differences in the associations between Shannon index and brain imaging measures after additional adjustment for education and BMI

| Imaging measures | *r*_males_ (*p*) | *r*_females_ (*p*) | *Z* value of sex comparison in *r* (*p*) | Cohen’s *q* |
| --- | --- | --- | --- | --- |
| GMV in R-Cbe Ⅵ | 0.409 (< 0.001) | 0.099 (0.405) | 2.058 (0.040) | 0.335 |
| CBF in B-CAL | 0.390 (< 0.001) | -0.228 (0.051) | 3.955 (< 0.001) | 0.644 |
| CBF in L-SFG | -0.463 (< 0.001) | -0.079 (0.501) | -2.592 (0.010) | 0.422 |
| AD | -0.620 (< 0.001) | 0.021 (0.857) | -4.582 (< 0.001) | 0.746 |
| RD | -0.496 (< 0.001) | 0.022 (0.853) | -3.476 (< 0.001) | 0.566 |
| MD | -0.483 (< 0.001) | 0.038 (0.746) | -3.470 (< 0.001) | 0.565 |

Abbreviations: BMI, body mass index; GMV, gray matter volume; CBF, cerebral blood flow; AD, axial diffusivity; RD, radial diffusivity; MD, mean diffusivity; Cbe Ⅵ, cerebellum Ⅵ; CAL, calcarine sulcus; SFG, superior frontal gyrus; R, right; B, bilateral; L, left.

**Table S3.** Sex differences in the associations between Chao index and brain imaging measures after additional adjustment for education and BMI

| Imaging measures | *r*_males_ (*p*) | *r*_females_ (*p*) | Z value of sex comparison in *r* (*p*) | Cohen’s *q* |
| --- | --- | --- | --- | --- |
| FCS in R-PCL | 0.508 (< 0.001) | -0.087 (0.467) | 3.976 (< 0.001) | 0.647 |
| FCS in L-PCL | 0.511 (< 0.001) | -0.126 (0.287) | 4.243 (< 0.001) | 0.691 |
| RD | -0.563 (< 0.001) | -0.031 (0.791) | -3.724 (< 0.001) | 0.606 |
| MD | -0.550 (< 0.001) | -0.012 (0.917) | -3.725 (< 0.001) | 0.606 |

Abbreviations: BMI, body mass index; FCS, functional connectivity strength; RD, radial diffusivity; MD, mean diffusivity; PCL, paracentral lobule; R, right; L, left.
